# Characterization of the complete chloroplast genome of an annual herb, *Chenopodium album* (Amaranthaceae)

**DOI:** 10.1080/23802359.2021.1920493

**Published:** 2021-06-30

**Authors:** Fu-Zhen Li, Xiao-Li Zhang, Ling-Long Zhu, Hong-fa Lu, Du-Lin Song

**Affiliations:** a Institute of Crop and Nuclear Technology Utilization, Zhejiang Academy of Agricultural Sciences, Hangzhou, P. R. China; b Hangzhou Campus, Zhejiang Normal University, Hangzhou, P. R. China

**Keywords:** *Chenopodium album*, chloroplast genome, phylogenetic analysis, Amaranthaceae

## Abstract

*Chenopodium album* is an annual herb from Amaranthaceae with worldwide distribution. It is a leafy vegetable as well as an important subsidiary grain crop with high nutritional value and medicinal value. In this study, we reported the complete chloroplast genome of *C. album*. The total chloroplast genome was 152,167 bp in length, containing a large single-copy region (LSC, 83,676 bp), a small single-copy region (SSC, 18,105 bp), and a pair of inverted repeat regions (IRs, 25,193 bp). The complete chloroplast genome contains 110 genes, including 78 protein-coding genes, 28 transfer RNA (tRNA) genes, and 4 ribosomal RNA (rRNA) genes with an overall GC content of 37.3%. Phylogenetic analysis showed that *C. album* was sister to *C. acuminatum* within Chenopodioideae. The complete chloroplast genome of *C. album* will provide useful resources for the development and utilization of this species and the phylogenetic study of Amaranthaceae.

*Chenopodium album* belongs to the complex genus *Chenopodium* of the family Amaranthaceae with worldwide distribution. It has been cultivated as a leafy vegetable as well as an important subsidiary grain crop for human and animal food-stuff due to its high-protein and balanced amino-acid spectrum with high lysine and methionine contents (Prakash and Pal 1998; Bhargava et al. 2003). In addition, *C. album* is also a traditional medicinal plant which can be used to treat peptic ulcer, dyspepsia, flatulence, strangury, pharyngopathy, splenopathy, opthalmopathy and general debility (Jabbar et al. 2007; Yadav et al. 2007). In recent years, interest in *C. album* as a valuable food source has renewed in Asia because of its versatility and wide adaptability (Poonia and Upadhayay 2015). However, few studies have been focused on its phylogeny, and its taxonomic status is still controversial (Hong et al. 2017; Yao et al. 2019). To better understand its evolution, here we characterized the complete chloroplast genome of *C. album* and presented a phylogeny of Amaranthaceae.

Fresh leaves of *C. album* plant were collected from Haining City (Zhejiang, China; 121°81′N, 30°78′E), and the voucher specimen (FZL_2020) was deposited at Institute of Crop and Nuclear Technology Utilization, Zhejiang Academy of Agricultural Sciences. Total genomic DNA was extracted from sampled leaves using the Plant Genomic DNA Kit (Tiangen Biotech, Beijing, China) following the manufacturer’s instructions. Then, raw reads were obtained by next-generation sequencing, conducting on the Illumina Hiseq Platform (Illumina, San Diego, CA). The complete chloroplast genome was assembled via NOVOPlasty (Dierckxsens et al. 2017) with the chloroplast genome sequence of *Chenopodium quinoa* (GenBank accession number: MK159176) as a reference sequence.

The annotation was performed using an online Dual Organellar GenoMe Annotator tool (Wyman et al. 2004). Finally, we obtained a complete chloroplast genome of *C. album*, which has been submitted to GenBank with accession number MW417304.

The complete chloroplast genome of *C. album* is 152,167 bp in length, consists of a large single-copy region (LSC, 83,676 bp), a small single-copy region (SSC, 18,105 bp), and a pair of inverted repeat regions (IRs, 25,193 bp). The complete chloroplast genome contains 110 genes, including 78 protein-coding genes, 28 transfer RNA (tRNA) genes, and 4 ribosomal RNA (rRNA) genes. The GC content of the chloroplast genome of *C. album* was 37.3%. Among these genes, 18 genes are duplicated in the IR regions, including all four rRNA genes, seven tRNA genes, and seven protein-coding genes.

To investigate the phylogenetic position of *C. album*, a phylogenetic analysis was performed based on the complete chloroplast genome sequences of 21 Amaranthaceae species. A maximum likelihood (ML) tree was reconstructed to determine the phylogenetic placement of *C. album* using PhyML v3.0 (Guindon et al. 2010), including tree robustness assessment using 100 rapid bootstrap replicates and with the GTRGAMMA substitution model, based on alignment of 66 shared protein-coding genes using MUSCLE v3.8.31 (http://www.drive5.com/muscle) (Edgar 2004). The results showed that *C. album* was sister to *C. acuminatum* with 99% bootstrap support value (Figure 1).

**Figure 1.**
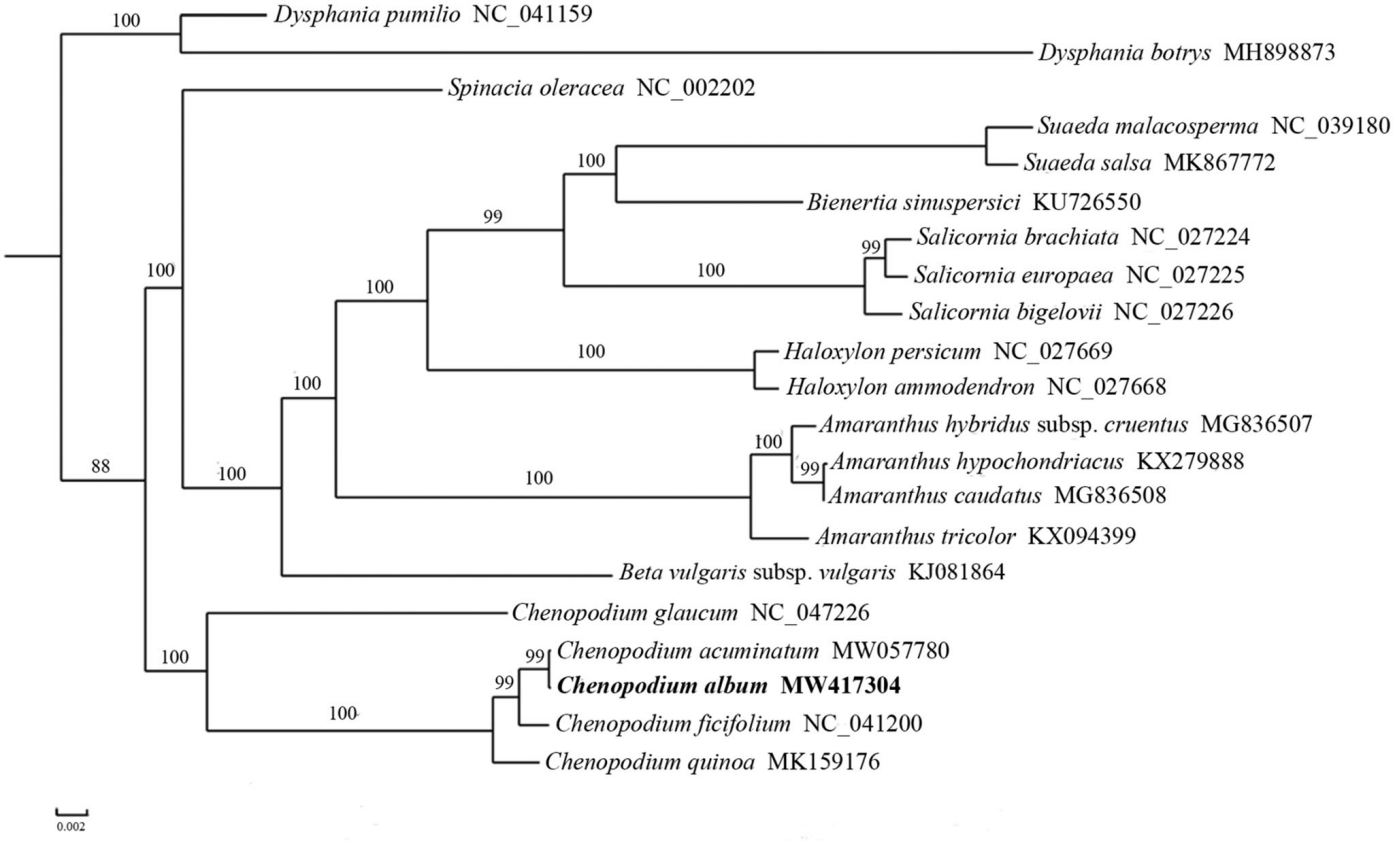
A maximum-likelihood (ML) tree inferred from 66 chloroplast genome genes. Two *Dysphania* species from Amaranthaceae are used as outgroup. The numbers on branches are bootstrap support values.

## Data Availability

The genome sequence data that support the findings of this study are openly available in GenBank of NCBI at (https://www.ncbi.nlm.nih.gov/) under the accession no. MW417304. The associated BioProject, SRA, and Bio-Sample numbers are BioProject, Bio-Sample and SRA numbers are PRJNA687826, SAMN17393254, and SRR13494532, respectively.

## References

[CIT0001] Bhargava A, Shukla S, Ohri D. 2003. Genetic variability and heritability of selected traits during different cuttings of vegetable *Chenopodium*. Ind J Genet Plant Breed. 63:359–360.

[CIT0002] Dierckxsens N, Mardulyn P, Smits G. 2017. NOVOPlasty: de novo assembly of organelle genomes from whole genome data. Nucleic Acids Res. 45(4):e18. 28204566 10.1093/nar/gkw955 PMC5389512

[CIT0003] Edgar RC. 2004. MUSCLE: multiple sequence alignment with high accuracy and high throughput. Nucleic Acids Res. 32(5):1792–1797. 15034147 10.1093/nar/gkh340 PMC390337

[CIT0004] Guindon S, Dufayard JF, Lefort V, Anisimova M, Hordijk W, Gascuel O. 2010. New algorithms and methods to estimate maximum-likelihood phylogenies: assessing the performance of PhyML 3.0. Syst Biol. 59(3):307–321. 20525638 10.1093/sysbio/syq010

[CIT0005] Hong SY, Cheon KS, Yoo KO, Lee HO, Cho KS, Suh JT, Kim SJ, Nam JH, Sohn HB, Kim YH. 2017. Complete chloroplast genome sequences and comparative analysis of *Chenopodium quinoa* and *C. album*. Front Plant Sci. 8:1696. 29056940 10.3389/fpls.2017.01696 PMC5635682

[CIT0006] Jabbar A, Zaman MA, Iqbal Z, Yaseen M, Shamim A. 2007. *Chenopodium album* (L.) an *Caesalpinia crista* (L.) against trichostrongylid nematodes of sheep. J Ethnopharmacol. 114(1):86–91. 17826017 10.1016/j.jep.2007.07.027

[CIT0007] Poonia A, Upadhayay A. 2015. *Chenopodium album* Linn: review of nutritive value and biological properties. J Food Sci Technol. 52(7):3977–3985. 26139865 10.1007/s13197-014-1553-x PMC4486584

[CIT0008] Prakash D, Pal M. 1998. *Chenopodium*: seed protein, fractionation and amino acid composition. Int J Food Sci Nutr. 49(4):271–275.

[CIT0009] Wyman SK, Jansen RK, Boore JL. 2004. Automatic annotation of organellar genomes with DOGMA. Bioinformatics. 20(17):3252–3255. 15180927 10.1093/bioinformatics/bth352

[CIT0010] Yadav N, Vasudeva N, Singh HS, Sharma SK. 2007. Medicinal properties of genus *Chenopodium* Linn. Nat Prod Radiance. 6:131–134.

[CIT0011] Yao Y, Li XT, Wu XY, Fan SJ, Zhang XJ, Qu XJ. 2019. Characterization of the complete chloroplast genome of an annual halophyte, *Chenopodium glaucum* (Amaranthaceae). Mitochondrial DNA Part B. 4(2):3898–3899. 33366241 10.1080/23802359.2019.1687041 PMC7707789

